# Chemical and Morphological Transition of Poly(acrylonitrile)/Poly(vinylidene Fluoride) Blend Nanofibers during Oxidative Stabilization and Incipient Carbonization

**DOI:** 10.3390/nano10061210

**Published:** 2020-06-21

**Authors:** Martin Wortmann, Natalie Frese, Al Mamun, Marah Trabelsi, Waldemar Keil, Björn Büker, Ali Javed, Michael Tiemann, Elmar Moritzer, Andrea Ehrmann, Andreas Hütten, Claudia Schmidt, Armin Gölzhäuser, Bruno Hüsgen, Lilia Sabantina

**Affiliations:** 1 Faculty of Engineering and Mathematics, Bielefeld University of Applied Sciences, Interaktion 1, 33619 Bielefeld, Germany; al.mamun@fh-bielefeld.de (A.M.); marah.trabelsi@enis.tn (M.T.); andrea.ehrmann@fh-bielefeld.de (A.E.); bruno.huesgen@fh-bielefeld.de (B.H.); lilia.sabantina@fh-bielefeld.de (L.S.); 2 Faculty of Physics, Bielefeld University, Universitätsstraße 25, 33615 Bielefeld, Germany; nfrese@uni-bielefeld.de (N.F.); bjoern.bueker@uni-bielefeld.de (B.B.); ahuetten@uni-bielefeld.de (A.H.); ag@uni-bielefeld.de (A.G.); 3 Ecole Nationale d’Ingénieurs de Sfax, University of Sfax, Route Soukra Km 3.5 B.P. 1173, Sfax 3038, Tunisia; 4 Department of Chemistry, Paderborn University, Warburger Straße 100, 33098 Paderborn, Germany; keilw@campus.uni-paderborn.de (W.K.); ali.javed@uni-paderborn.de (A.J.); michael.tiemann@uni-paderborn.de (M.T.); claudia.schmidt@uni-paderborn.de (C.S.); 5 Faculty of Mechanical Engineering, Paderborn University, Warburger Straße 100, 33098 Paderborn, Germany; elmar.moritzer@uni-paderborn.de

**Keywords:** electrospinning, carbon nanofiber, polymer blend, stabilization, carbonization, poly(acrylonitrile) (PAN), poly(vinylidene fluoride) (PVDF)

## Abstract

Thermally stabilized and subsequently carbonized nanofibers are a promising material for many technical applications in fields such as tissue engineering or energy storage. They can be obtained from a variety of different polymer precursors via electrospinning. While some methods have been tested for post-carbonization doping of nanofibers with the desired ingredients, very little is known about carbonization of blend nanofibers from two or more polymeric precursors. In this paper, we report on the preparation, thermal treatment and resulting properties of poly(acrylonitrile) (PAN)/poly(vinylidene fluoride) (PVDF) blend nanofibers produced by wire-based electrospinning of binary polymer solutions. Using a wide variety of spectroscopic, microscopic and thermal characterization methods, the chemical and morphological transition during oxidative stabilization (280 °C) and incipient carbonization (500 °C) was thoroughly investigated. Both PAN and PVDF precursor polymers were detected and analyzed qualitatively and quantitatively during all stages of thermal treatment. Compared to pure PAN nanofibers, the blend nanofibers showed increased fiber diameters, strong reduction of undesired morphological changes during oxidative stabilization and increased conductivity after carbonization.

## 1. Introduction

The production of carbon nanofibers (CNFs) based on carbonized electrospun nanofibers has received increasing attention in recent years, owing to the cost efficiency of electrospinning and ongoing development of new commercial applications [1,2]. Due to the special properties of one-dimensional nanomaterials, such as nanofibers, nanowires and nanotubes, there is a great potential for application in nanocomposites [3,4], filtration [5,6,7], batteries [8,9,10], superconductors [11], nano-electronics [12] or tissue engineering [13,14,15]. There are now several different electrospinning methods for the production of CNFs. These include needle-, cylinder- or wire-based electrospinning from polymer solutions or melts, each with their respective advantages and disadvantages [16,17,18,19,20]. Needleless (wire-based) electrospinning is usually credited with the greatest potential for the large-scale production nanofibers [16]. Numerous studies have dealt with the investigation of process parameters, precursor polymers or additives such as nanoparticles and their influence on the properties of the resulting CNFs [19,20].

Poly(acrylonitrile) (PAN) is a commonly used precursor for the production of CNFs via electrospinning followed by oxidative stabilization and carbonization [21,22]. Oxidative stabilization is a heat treatment process during the production of carbon fibers. It prevents melting or fusing of the fibers and minimizes the volatilization of elemental carbon to maximize the final carbon yield in the subsequent carbonization process [23]. Both stabilization and carbonization have a significant influence on fiber morphology, composition and resulting properties and thus decide on potential applications [24,25]. The stabilization in air usually involves a combination of cyclization, dehydrogenation, oxidation, aromatization and crosslinking reactions resulting in aromatic ladder-structure [26,27,28]. The subsequent carbonization to produce CNFs usually takes place in a nitrogen atmosphere, more rarely also in argon [29,30], and produces different chemical and morphological properties depending on the treatment temperature. Both the carbonization process and the properties of CNFs have been reported in detail in the literature [31,32,33,34,35]. While some studies showed the influence of different nanoparticles on CNFs [36,37,38,39], only few dealt with the carbonization of blend nanofibers from two or more different polymers. Their use as precursors for carbonization can serve different purposes. The main incentive is usually a morphological modification of the resulting CNF where one polymer is completely removed from the fibers either before thermal treatment by dissolution or during thermal treatment by pyrolysis [40,41,42,43]. Another incentive is to implement persistent ingredients such as nitrogen [44], phosphorus [45] or nanofillers and particles [36,46] into the CNF to improve electrical or mechanical properties for instance. Depending on the desired field of application, this can likewise be achieved by post-treatment after carbonization by various methods [47,48,49,50]. The use of blend nanofibers produced by electrospinning binary polymer solutions can be an effective alternative route to producing differently doped carbon nanofibers with adjustable fiber morphology. In the field of energy storage where CNFs are investigated, for example, as anode material for lithium-ion batteries, a method that can potentially be used for large-scale production to improve the electrical properties, such as the carbonization of blend fibers, is of particular interest [51,52,53,54,55,56]. Postproduction fluorine doping of CNFs has already shown to be a promising method to increase electrochemical properties in capacitors, gas-sensors and metal-free electro-catalyst applications [50,57,58].

Due to its good electrochemical stability and affinity to electrolyte solutions, poly(vinylidene fluoride) (PVDF) has recently received significant attention for its potential as a precursor for CNFs and their potential application in lithium-ion batteries [59,60,61,62]. While Yang et al. emphasized the need for low temperature chemical stabilization by dehydrofluorination for the subsequent carbonization of PVDF [61], other groups have shown that carbonization under a nitrogen atmosphere is possible without additional stabilization [59,63,64], suggesting that carbonizing PAN/PVDF may enable creating carbon fibers combining the advantages of both materials. PVDF has also been studied as electrospun blend membrane with PAN [65].

Although the chemical processes involved in the stabilization and carbonization of pure PAN have been studied for over 60 years, practically nothing is known about the mechanisms in blend fibers and the interaction of two or more polymers in them. The application of such blend fibers is extremely promising and an obvious upgrade for many applications of nanofibers. A major reason why the advantages of blend fibers are rarely utilized in functional applications is that the basic processes are less well understood.

In this work, we report on the preparation, carbonization and resulting properties of PAN/PVDF blend nanofibers produced by wire-based electrospinning of binary polymer solutions in DMSO. This blend is of high interest since opposite to blending PAN with gelatin or other biopolymers, which degrade below typical stabilization temperatures [66], it offers the possibility to modify the carbon fiber morphology without losing too much material and thus strongly reducing the overall carbon yield. Due to the aforementioned potential of PVDF as a precursor of carbon nanofibers especially for lithium-ion batteries and the well-known high carbon yield of PAN, it is natural to study a blend of these two materials. A comprehensive investigation of the chemical and morphological transition was performed to deepen the understanding of established stabilization and carbonization routes for blend nanofibers. Through the broad use of different characterization methods, we would also like to address problems and limitations of various measurement methods for nanofibers in a broader context.

## 2. Materials and Methods

### 2.1. Sample Preparation

Nanofibers were produced by the wire-based electrospinning machine Nanospider Lab (Elmarco Ltd., Liberec, Czech Republic) on a polypropylene nonwoven collector substrate. The following spinning parameters were used for the production of nanofibers: high voltage of 60 kV, resulting in a typical current of 0.03 mA; nozzle diameter of 0.9 mm; carriage speed of 100 mm/s; distance from electrode to collector 240 mm; distance from ground electrode to collector 50 mm; process time 20 min; and temperature 24 °C and relative humidity 32% in the spinning chamber.

Three different polymer solutions of PAN (CAS No. 25014-41-9, copolymer with 6% methyl methacrylate, Dralon, Dormagen, Germany) and PVDF (CAS No. 24937-79-9, Ambofluor, Hamburg, Germany) in DMSO (CAS No. 67-68-5, 99.9%, S3 Chemicals, Bad Oeynhausen, Germany) were used for electrospinning: (a) a relatively low polymer concentration of 12 wt% PAN and 3 wt% PVDF (referred to as PAN 4:1 PVDF); (b) a relatively high polymer concentration of 15 wt% PAN and 5 wt% PVDF (referred to as PAN 3:1 PVDF); and (c) a reference of 16 wt% PAN (referred to as PAN), which is a commonly used concentration for electrospinning PAN from DMSO. These concentrations are the result of preliminary trials and have been selected according to their apparent spinnability. The blend ratios were chosen based on prior experiments, showing that PAN:PVDF blends of 1:1 or 2:1 were not or hardly spinnable, while higher PAN contents resulted in nanofiber mats quite similar to pure PAN.

The nanofibers were stabilized in a muffle furnace B150 (Nabertherm, Lilienthal, Germany), approaching a typical stabilization temperature of 280 °C at a heating rate of 1 °C/min in air, followed by isothermal treatment for 1 h. This procedure was found well suited for oxidative stabilization of pure PAN nanofibers [23]. For subsequent carbonization, a furnace (Carbolite Gero, Neuhausen, Germany) was used, approaching a temperature of 500 °C with a heating rate of 10 °C/min in a nitrogen (CAS No. 7727-37-9, ≥99.999%, Linde, Pullach, Germany) flow of 150 mL/min (STP), followed by isothermal treatment for 1 h. While this temperature is too low to reach full carbonization, it is well above the degradation temperature of the pure PVDF powder used in this study, as shown in Section 3.1, thus giving rise to possible morphological modifications related to degradation of pure PVDF fiber parts.

### 2.2. Characterization Methods

Morphological investigations of the nanofibers were performed with a confocal laser-scanning microscope (CLSM) VK-8710 (with maximal resolution of about 250 nm; Keyence Deutschland, Neu-Isenburg, Germany) and by a helium-ion microscope (HIM) Orion Plus (with a resolution between 0.75 and 0.25 nm; Carl Zeiss, Oberkochen, Germany). HIM images were taken at an acceleration voltage of 34.8 kV and a current of 1.2 pA. For charge compensation in secondary electron detection, an electron flood gun was used during imaging.

The amplitude-sensitive eddy current method was used to measure the thickness of the nonwovens with a FISCHERSCOPE MMS PC2 from Helmut Fischer (Berlin, Germany). The current in a coil around a ferrite core of the probe generates a high-frequency magnetic field, which induces eddy currents in the substrate. Their strength depends on the distance between the probe and the substrate, i.e., the thickness.

Thermo-gravimetric analysis (TGA) and differential scanning calorimetry (DSC) were carried out with a Hi-Res TGA 2950 Thermo-gravimetric Analyzer from TA Instruments (New Castle, DE, USA) and a DSC 3 device by Mettler-Toledo (Gießen, Germany), respectively. Both heating rates and atmospheres followed the aforementioned stabilization and carbonization procedure.

A 300-MHz Apollo spectrometer by Tecmag (Houston, TX, USA) equipped with a wide-bore Oxford magnet was used for ^13^C MAS NMR measurements. An magic angle spinning (MAS) probe for rotors with a diameter of 4 mm was used; the spinning frequency was 7 kHz. Data acquisition was carried out at 25 °C, using single pulse excitation and ^1^H decoupling. A spectral width of ±250 kHz, 2048 time domain data points, a 90° pulse duration of 3.5 μs, a recycling delay of 5 s and 2048 scans were used for ^13^C data acquisition. The adamantane signal at 29.5 ppm was used as an external chemical shift reference. The chemical shifts reported have an error of ±0.1 ppm.

Fourier transform infrared spectroscopy in attenuated total reflection (ATR-FTIR) was conducted on a FT/IR-4100 spectrometer by JASCO (Mary’s Ct, MD, USA) with a diamond ATR crystal, which results in a common penetration depth of about 1.7 µm, depending on angle and wavenumber. The spectra cover a frequency range from 4000 to 700 cm^−1^. Each spectrum was averaged over 32 scans and corrected for atmospheric noise.

X-ray photoelectron spectroscopy (XPS) was performed in an Omicron Multiprobe ultra high vacuum system (Scienta Omicron GmbH, Taunusstein, Germany) using monochromatic Al K_α_ irradiation, a Sphera electron analyzer with a resolution of 0.9 eV, and an emission angle of 20°. The probe area from which information is obtained is about 2 mm in diameter. Quantitative evaluation was based on peak integration in OriginPro2020. The atomic fraction was calculated from C 1s, O 1s, N 1s, and F 1s peak areas and their respective relative sensitivity factors published by Wagner et al. [67].

Investigations on the chemical composition of the treated and untreated nanofibers were performed with energy dispersive X-ray spectroscopy (EDX) in a FEI Helios Nanolab 600 dual beam focused ion beam electron microscope (FEI Deutschland GmbH, Frankfurt a. M., Germany). The machine is equipped with a multichannel analyzer to detect the characteristic X-ray spectrum emitted by the sample upon irradiation with high-energy electrons. The measurements were performed on an area of 200 µm × 200 µm at an acceleration voltage of 20 kV, which is sufficient to pick up the K_α_ peaks of light elements such as carbon, oxygen, nitrogen and fluorine. Additionally, the X-ray signal was mapped to a secondary electron image of individual blend nanofibers generated by the Helios Nanolab 600. For this purpose, the nonwovens were glued to a silicon wafer via conductive carbon tape, sputtered with a 10 nm gold layer and mounted on the sample stage.

Impedance measurements were conducted with a Novocontrol broadband dielectric spectrometer (Alpha-A High Performance Frequency Analyzer). The carbonized nonwovens were cut into small discs of 20 mm diameter and placed between two gold electrodes in a Novocontrol BDS1200 sample cell. The mounting stage of the cell was removed and the electrodes were connected to BNC cables through custom-built electrical connections. The BNC cables were connected to the analyzer through a Novocontrol ZG4 test interface (two-wire mode). The temperature was controlled by an Espec SH-242 climate chamber. A 100 Ω reference sample (Novocontrol) was used to test the connections.

## 3. Results

### 3.1. Morphology

From a broad span of spinnable PAN/PVDF polymer solutions, two concentration ratios between PAN and PVDF were chosen, 3:1 and 4:1, respectively. Since the carbonization of PAN fibers has been intensively investigated since the 1950s and is very well understood today, PAN is considered the reference polymer in this study. The aim was to investigate the chemical and morphological influences of blending with a second polymer (PVDF). Pure PAN nanofibers spun from a 16 wt% solution served as a reference. While there are many different methods with regard to process gases, heating rates and maximum temperatures, we opted for a well-established process route.

**Figure 1:**
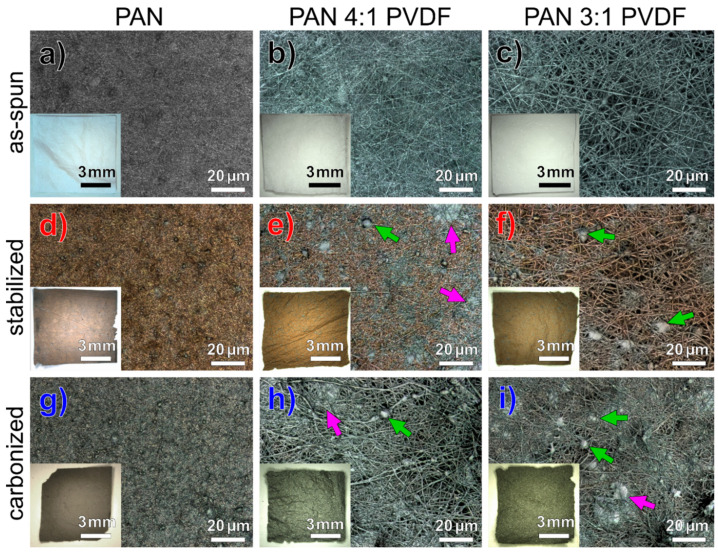
CLSM images and inserted photographs of (**a**–**c**) as-spun, (**d**–**f**) stabilized and (**g**–**i**) carbonized nanofiber nonwovens. Arrows indicate agglomerates (green) and membranous areas (purple).

First, the macroscopic appearance of the nanofibers deposited as nonwovens was examined. Figure 1 shows photographs of 1 cm × 1 cm nanofiber nonwoven and the respective CLSM images with a 2000x magnification. As usual, the fibers show a tan-brown discoloration when stabilized and a black discoloration with incipient carbonization. Clear differences between the various nanofiber nonwovens can already be seen with the naked eye. The CLSM images show a significant increase in the fiber diameter due to the PVDF modification (Figure 1a–c). Although too thin to see at this magnification, the as-spun nanofibers still appear largely homogeneous. Different phases form during stabilization, which can also be recognized by different discoloration in the CLSM, i.e., brown color shown by PAN fibers, while silvery areas can be identified as PVDF [66]. This can be attributed to the only partial miscibility of PAN and PVDF [68]. In some cases, agglomerates and membranous areas arise that cannot be seen in the pure PAN nonwoven. This also leads to color differences in the macroscopic appearance as seen in the respective photographs.

**Figure 2:**
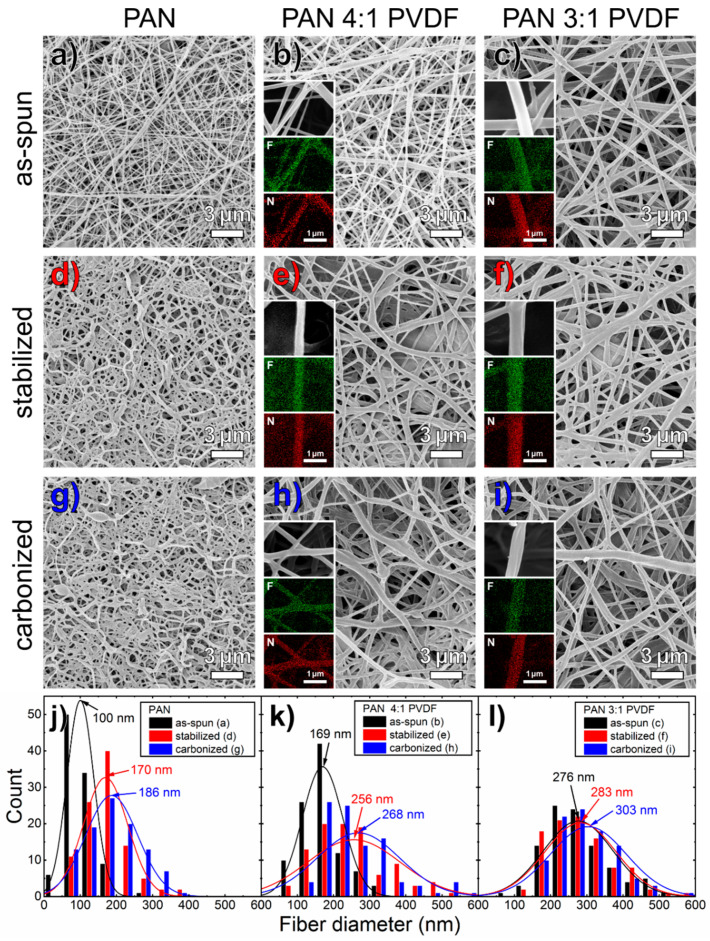
(**a**–**i**) HIM images of all examined nonwovens with a field of view of 20 µm × 20 µm in combination with EDX maps on individual blend nanofibers, taken at the same samples and given as inserts for fluorine (green) and nitrogen (red) signal images, respectively; and (**j**–**l**) corresponding fiber diameter histograms with normal distributions.

The HIM images in Figure 2 confirm the significant increase in fiber diameter not only through the PVDF modification, but also through oxidative stabilization. These phenomena have been investigated in numerous studies. The morphological change upon stabilization follows from the release of internal tension in the fiber, which was introduced during electrospinning due to the extreme stretching of the polymers—both shrinkage in the fiber direction and fiber diameter increase.

Although the CLSM images in Figure 1e,f indicate a partial separation of the polymer phases, the EDX maps in Figure 2 clearly show that these are actual blend nanofibers containing both polymers or rather, after thermal treatment, both fluorine and nitrogen. As Figure 1h,i show, the agglomerates formed during stabilization partially persist during carbonization. Since pure PAN usually turns tan-brown due to stabilization, as Figure 1d shows, it can be assumed that the areas that appear grey are mainly composed of PVDF. This behavior was also reported in [66] for PAN/gelatin blend nanofiber nonwovens that similarly showed some silvery nanofibers after stabilization, which consisted mainly of gelatin, while the fibers with average or predominant PAN content showed the usual tan-brown color.

The HIM images show that all as-spun nanofibers are relatively straight, have very few membranous regions and show typical beads, which are often observed in electrospinning with DMSO or other slowly evaporating solvents [69,70]. Figure 2a,d,g show that the pure PAN fibers experience a clear morphological change towards more reticular and strongly contorted fibers during stabilization, which, as previous studies have shown, can be prevented by fixing the fibers on a robust substrate [71] or stretching during treatment [72,73]. The PVDF-modified fibers, on the other hand, do not experience any significant morphological change upon both stages of thermal treatment apart from a slight increase in fiber diameter. This indicates that an increased diameter improves the resistance of the fibers to contraction, thus sustaining the overall shape of the nonwoven. Pure PAN shows, as usual, a certain number of beads in the fibers, which is known to depend on various process parameters. Except for a few fiber knots, the PVDF-modified fibers contain no visible beads. The inhomogeneity shown in Figure 1e,f,h,i is also reflected in a broadening of fiber diameter distribution of the PVDF-modified fibers. In general, it can be stated that the addition of PVDF contributes to maintaining the geometric structure of the fibers during stabilization and carbonization. For all samples examined, the fiber topology appears to be mostly smooth.

A comparison of the CLSM and HIM images shows that highly magnified images, which are common in this field to examine the fiber topology and diameter distribution, lack essential information about the homogeneity on a more macroscopic scale. Additional high-resolution images take time and effort and are often rejected or at least not demanded by reviewers. Therefore, this is a problem that is often not sufficiently addressed in the literature or is dismissed with the undocumented assurance of representativeness of micrographs, bringing along reproducibility issues [74]. The comparison of different microscopy methods emphasizes the benefits of light microscopy, especially with regard to large-scale applications of the electrospinning process. In addition, relevant color information can be obtained, and, moreover, the representativeness is demonstrated with a lower magnification.

**Figure 3:**
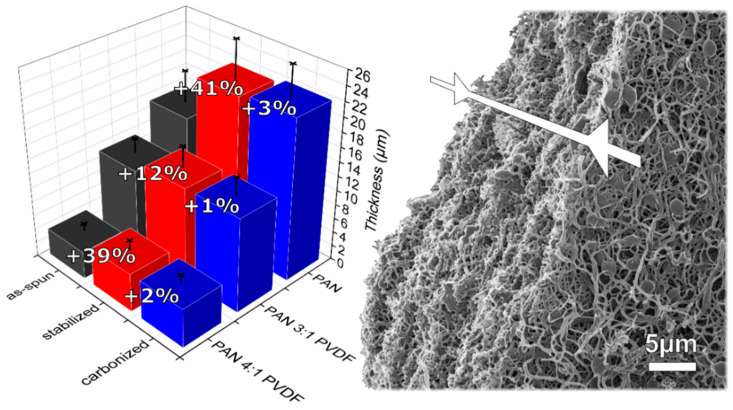
Results of thickness measurements by the amplitude-sensitive eddy current method of all examined nonwovens with respective percentage changes from one process step to the next and HIM image of a carbonized PAN nonwoven edge as seen from diagonally above. Exact thickness values are given in Table 1.

Despite an increase in the fiber diameter, a reduction in the thickness of the nonwoven fabric is observed, as shown in Figure 3. This also indicates that the PVDF modification leads to a reduction in the total output of the spinning process (weight per area and second), which can also be inferred from the reduced nonwoven thickness in relation to the same process time. The 3:1 sample has a higher total polymer concentration in the solution, but the resulting nonwoven thickness is reduced. This is in agreement with previous observations that lower concentrations generally result in a higher mass yield during identical spinning durations, while higher concentrations lead to a reduced mass yield but more separated fibers without membranous regions [75]. Apart from that, the comparison with the 4:1 sample shows that, as usual, the fiber diameter (within a reasonable range of process parameters) increases with the polymer concentration in the spinning-solution. Interestingly, both the thicknesses of the fibers and of the nonwovens only increase significantly during stabilization. The subsequent carbonization showed no significant morphological change in any of the samples. This effect has been observed before for stabilized PAN nanofiber nonwovens [70].

### 3.2. Polymer Composition

**Figure 4:**
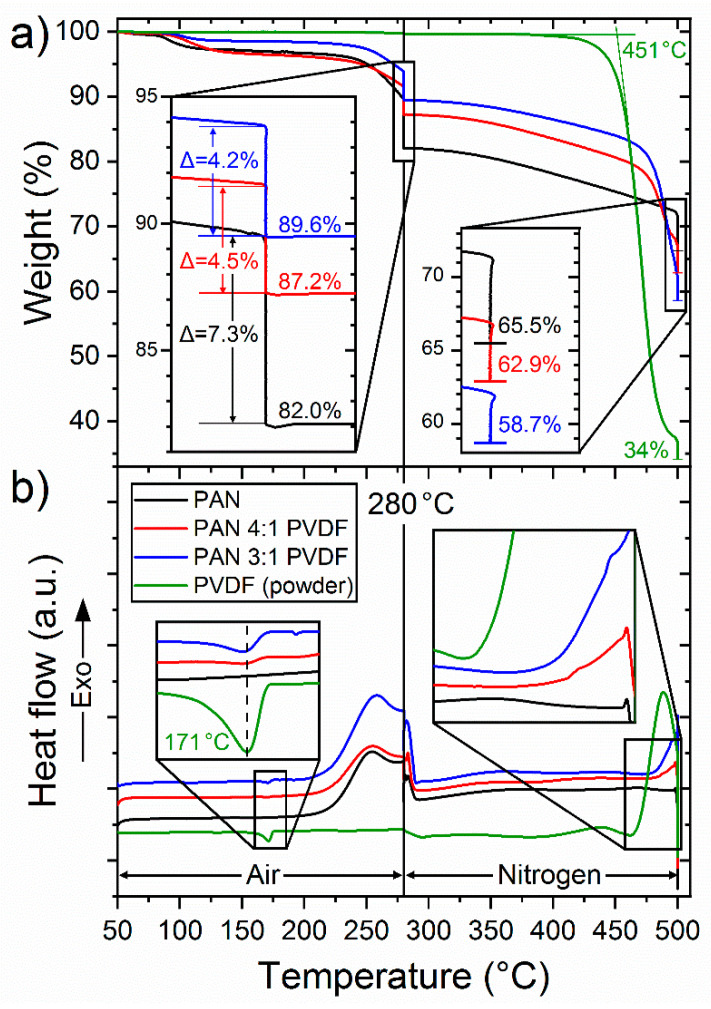
Results of (**a**) TGA and (**b**) DSC measurements of all examined nanofiber nonwovens according to the described stabilization and carbonization routes. The discontinuities in the curves indicate 1 h isothermal phases. Note that the highlighted numbers show remaining mass values.

Figure 4a shows the weight loss curves during stabilization and carbonization. The initial small decrease in mass of the nanofiber samples of about 3% at approximately 85 °C is mostly due to moisture and solvent residues, which can also be seen in the FTIR spectra of as-spun nanofibers. However, pure PVDF powder has an extremely low moisture absorption due to its hydrophobic and semi-crystalline character and therefore shows no weight loss until the onset of thermal decomposition at about 410 °C, which is in good agreement with the literature [76]. The fact that PVDF shows no weight loss whatsoever up to 280 °C therefore explains why the weight loss of the nanofiber samples increases at the end of the isothermal stabilization phase with decreasing PVDF content.

**Figure 5:**
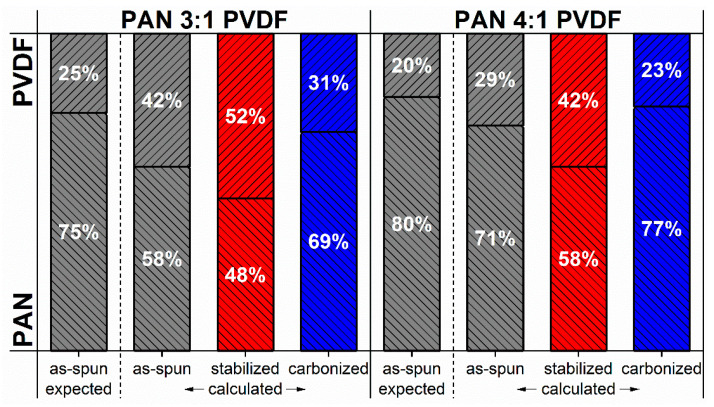
Calculated polymer mass ratios of blend nanofibers based on TGA measurements compared to the mass ratios that would be expected due to the polymer concentrations in the spinning-solutions.

The polymer concentration in the spinning-solution is not necessarily representative of the polymer concentration in the resulting blend nanofiber nonwoven. In fact, as shown in Figure 5, this can be deduced from the TGA measurements, since the mass of PVDF is constant during stabilization and the mass loss of pure PAN is known (82%). For the 3:1 as-spun sample, it results a PAN concentration of about 58%, which decreases to about 48% during stabilization. For the 4:1 samples, the respective PAN concentrations are 71% before and 58% after stabilization. Since the PVDF loses 66% of its weight during the carbonization phase due to thermal decomposition (significantly more than pure stabilized PAN), the final mass loss for the PVDF-modified fibers is greatest. The order of mass losses after stabilization is thus reversed after carbonization: 34.5% for pure PAN, 37.1% for 3:1 and 41.3% for 4:1 nanofibers.

Since the mass losses during carbonization of pure stabilized PAN of 16.5% and pure PVDF of 66% are now known, the contribution of the two polymers to the resulting total residual mass can be calculated, as shown in Figure 5.

Since TGA measurement curves as well as the quality of thermal treatment also depend on the sample geometry, which differs from the samples treated in the furnace, it is important to note that this can only be considered an approximation. Furthermore, these calculations do not take into account the geometry of the fibers or nonwovens, which could possibly have an influence on TGA measurements. How accurate this calculation method is should be determined by comparison with other quantitative measurement methods in further studies.

The aromatization of PAN is an exothermic process [27], which can be seen in the DSC curves in Figure 4b as exothermic peaks above 200 °C. The maximum heat development during this reaction depends on the heating rate. Here, with a heating rate of 1 °C/min in air, it is reached at about 255 °C, which is in good agreement with earlier studies [24].

DSC measurements have shown a crystallite melting peak temperature of 171 °C and a crystallinity of about 39% for pure PVDF powder, calculated as follows:

(1) $$\chi_{c}\left(t\right)=\Delta H_{m}/\Delta H_{100\%}$$

where χ_c_(t) is the actual crystallinity of the sample, ΔH_m_ (≈41 J/g) is the heat of melting, determined by integration of the crystallite melting peak and ΔH100% (≈105 J/g) is the heat of melting for a theoretical 100% crystalline PVDF sample [77]. The inset for low temperatures in Figure 4b shows that the PVDF-modified fibers also have a small endothermic peak in the DSC curve at the same temperature. The observation of melting peaks of PVDF also supports the partial segregation of the polymer phases, which can be seen from the color differences in the CLSM images. The normalized peak integrals of the 3:1 and 4:1 samples give a crystallinity of the PVDF phase with respect to the total sample mass of 7% and 4%, respectively. If one assumes the calculated polymer mass ratios of the as-spun blend fibers to be correct, values of 16% and 11% would be expected for a pure PVDF phase with a degree of crystallization of 39%. Not surprisingly, the crystallization of PVDF polymers in the blend fibers would thus be strongly restrained. To what extent the crystallinity of PVDF is affected by the spinning process or by the presence of the PAN polymers remains open. However, it is known that crystallization in electrospinning is generally reduced compared to the raw material due to the short time available to reconfigure the polymers [78].

The thermal decomposition of PVDF can be seen in the DSC curve as a steep exothermic peak towards the end of the carbonization phase. As the inset for high temperatures in Figure 4b shows, the exothermic decomposition begins somewhat delayed in the PVDF-modified fibers. This suggests that the PVDF polymers are at least in part thermally insulated inside the fibers. The decomposition of the PVDF polymer structure during carbonization leads to the formation of smaller reactive fragments, thus increasing diffusion mobility and enabling covalent integration of fluorine compounds into the now aromatic PAN matrix. This mechanism is examined in more detail below based on spectroscopic data.

### 3.3. Chemical Structure

**Figure 6:**
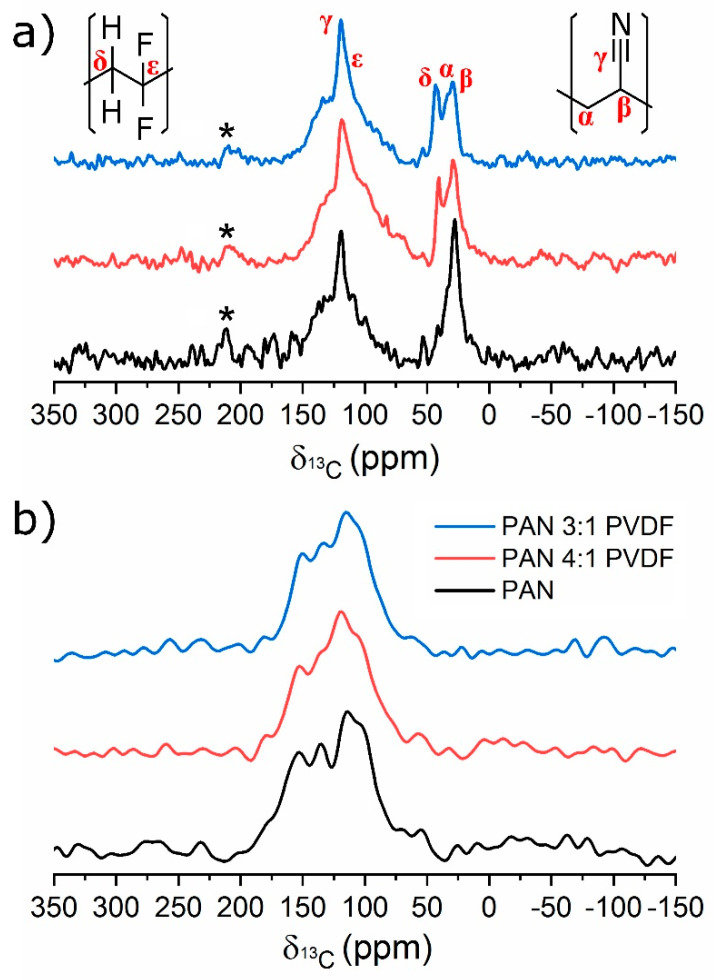
Solid-state ^13^C spectra obtained under MAS at 7 kHz of (**a**) as-spun and (**b**) carbonized nanofibers. The peaks labeled with a star are spinning sidebands recognized by their distance of 7 kHz with respect to the γ peak. Spinning sidebands result from magic angle spinning [82].

Figure 6 shows solid-state ^13^C NMR spectra of as-spun and carbonized nanofibers. These spectra are not fully relaxed and intensity ratios are not quantitative. The main peaks in the spectra of the as-spun samples, as shown in Figure 6a, can be assigned according to solution spectra of PAN [79,80] and PVDF [81]. The signal of the C≡N carbon atom (γ) occurs at approximately 120 ppm. The methine (β) and methylene (α) signals of PAN at about 35 and 30 ppm, respectively, are not resolved. For PVDF, two ^13^C NMR signals are expected. The one of the CH_2_ group (δ) is found as an additional peak at about 42 ppm, whereas the one of the CF_2_ group (ε) overlaps with the PAN resonance at 120 ppm and can be recognized as a broadening of the upper part of this signal. These results confirm that PVDF is incorporated into the as-spun nanofibers as expected.

As shown in Figure 6b, after carbonization, the chemical modification known for PAN is evident from the spectral changes in the region from 50–200 ppm. The broadness of the peaks is due to overlapping signals from a multitude of structures with slightly different chemical shift values. Wang et al. reported detailed solid-state NMR studies of heat-treated PAN [80]. They present numerous possible structures, which were correlated to ^13^C peaks between 100 and 160 ppm. All of these cyclic structures contain nitrogen atoms. Such highly cyclized structures, which contain nitrogen atoms, are probably present in the carbonized nanofibers. Note the absence of ^13^C signals in the aliphatic range between 0 and 50 ppm. This is a strong indication that PVDF is no longer present as a linear polymer after carbonization, at least not in larger quantities. The reason for that is probably that PVDF decomposes during thermal treatment. Due to the poor signal-to-noise ratio, potential differences between the three samples cannot be distinguished.

**Figure 7:**
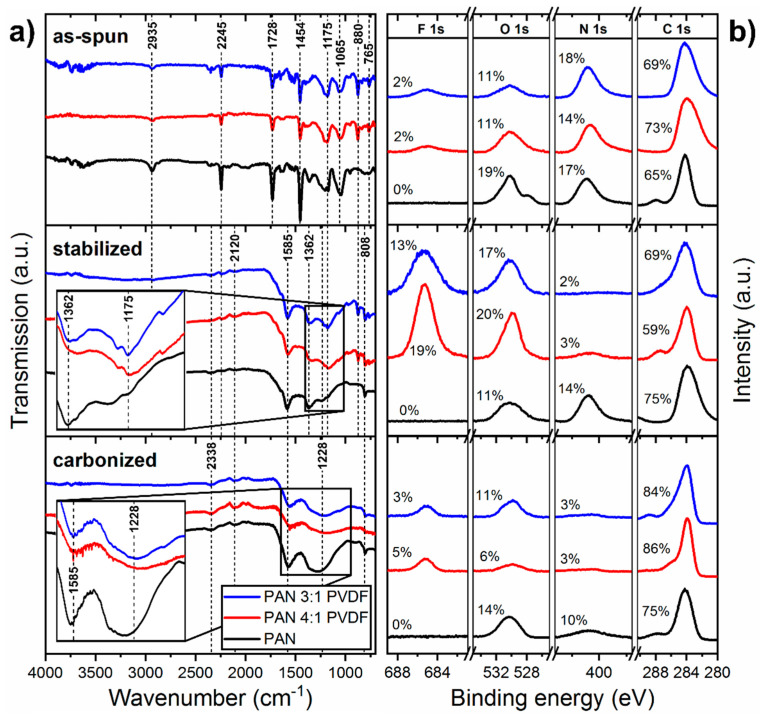
(**a**) ATR-FTIR and (**b**) XPS spectra of as-spun, stabilized and carbonized nanofibers. XPS spectra show F 1s, O 1s, N 1s and C 1s signals separately with calculated relative atomic ratios.

Figure 7a shows ATR-FTIR spectra of all as-spun, stabilized and carbonized samples. In all samples of as-spun nanofibers, the typical peaks of PAN are observable: a stretching vibration of the C≡N nitrile functional group at 2245 cm^−1^ and a carbonyl (C=O) stretching peak at 1728 cm^−1^, which is an indicator of both residual DMSO [83] and the methyl methacrylate comonomer. Furthermore, the bending and stretching vibrations of CH_2_ at 2935 and 1454 cm^−1^, respectively, can be seen [19]. In addition, the bands at 1175, 880 and 765 cm^−1^ represent the symmetrical stretching of the CF_2_ group, the asymmetrical stretching of C–C–C and the C–F stretching, respectively, that are commonly attributed to PVDF [47,84,85,86]. Therefore, the spectra show that both polymers are present in the as-spun blend nanofibers. After stabilization, the absorption increases strongly across the entire spectrum. The distinct nitrile peak at 2245 cm^−1^ and the CH_2_ peak at 2935 cm^−1^ disappear completely and new aromatic C=C, C=N imine and C–N heterocycle peaks at 1585, 1362 and 808 cm^−1^, respectively, emerge, clearly indicating the formation of a nitrogen containing ladder-structure [87]. The most apparent difference between the pure and PVDF-modified fibers is visible in the range between 1000 and 1400 cm^−1^ where the C–F stretching vibration is expected to show a strong, broad signal (region highlighted in Figure 7a) and especially at around 1175 cm^−1^ for the CF_2_ stretching vibration [88].

It is known that the FTIR spectrum becomes flatter as carbonization progresses and discrete peaks gradually disappear. In the spectra of the carbonized samples, the most apparent difference between pure and PVDF-modified fibers is that the broad merged peak between 1000 and 1400 cm^−1^ is considerably flatter for the blend fibers; a region usually associated with C=C, C–N and C=N peaks [89]. This could be an indication that the fusion of the ladder-polymers, usually associated with carbonization, is partially hindered by the presence of fluorine bonds. This seems plausible, since inert C–F bonds are not suitable for the inter-polymer reactions that usually take place between the opposite C–H and C–N=C containing sides of the ladder-structure during carbonization [31]. This reduced N:F ratio, which is suggested by the FTIR spectra, shows good agreement with the corresponding XPS spectra, where a steep increase in fluorine concentration in the fiber surface can be seen after stabilization.

FTIR and EDX have penetration depths in the micrometer range, which is suitable for obtaining information about the entire fiber diameter and possibly even several fiber layers. XPS measurements, as shown in Figure 7b, on the other hand are extremely surface-sensitive with a penetration depth of about 5 nm [89]. XPS on nanofiber nonwovens have proven to be very challenging. While carbonized nanofibers have sufficient conductivity, as-spun and stabilized nanofiber nonwovens sometimes have significant charging effects despite the use of an electron flood gun. The reason for this is that emitted photoelectrons cannot flow off in electrical insulators, similar to most polymers, without moving charge carriers. Apart from that, the fibrous structure is not as suitable for even charge distribution as homogeneous bulk material anyway. The result is a shift towards higher binding energies. A deformation of the peaks or artifacts can often be observed (see Figure 7b: as-spun PAN, O 1s). The charge effects were compensated here by charge referencing on the carbon peak. Another major challenge with XPS analysis of nanofiber nonwovens is that the samples react intensely with ubiquitous atmospheric carbon and oxygen due to their high specific surface area [89]. As a result, adventitious carbon is formed on the fiber surface, which mainly consists of polymeric hydrocarbon (C–C/C–H) and carbon oxides (C–O–C/O–C=O) [90]. As can also be seen in most of the C 1s spectra shown, this can lead to amalgamation of the aliphatic C–C/C–H peak with the O–C=O carbon oxide peak, which is by no means unproblematic with regard to charge referencing [91]. Since neither PAN nor PVDF contains oxygen, the O 1s peaks of the as-spun fibers can only be explained by the adsorption of atmospheric oxygen. Although the 6 wt% of methyl methacrylate comonomer in PAN contains oxygen, this cannot account for 11–19 at% of oxygen in the fiber surface, as indicated by XPS. It might, however, be one explanation for the difference between pure PAN fibers and blend fibers. The quantitative evaluation shows that the oxygen content increases with smaller fiber diameter, which might indicate a correlation with a higher specific surface.

These challenges mean that a qualitative evaluation of the surface chemistry through peak fitting is highly unreliable. Instead, special attention was paid to a quantitative evaluation. Although the peak signal intensity relative to the background should be less affected by the problems mentioned, outliers can also be observed here (see Figure 7b: stabilized 4:1, F 1s). Since the surface of a nanofiber nonwoven is very heterogeneous on small scales, it is generally recommended to use the largest possible analysis area in order to be able to obtain an average over local differences.

**Figure 8:**
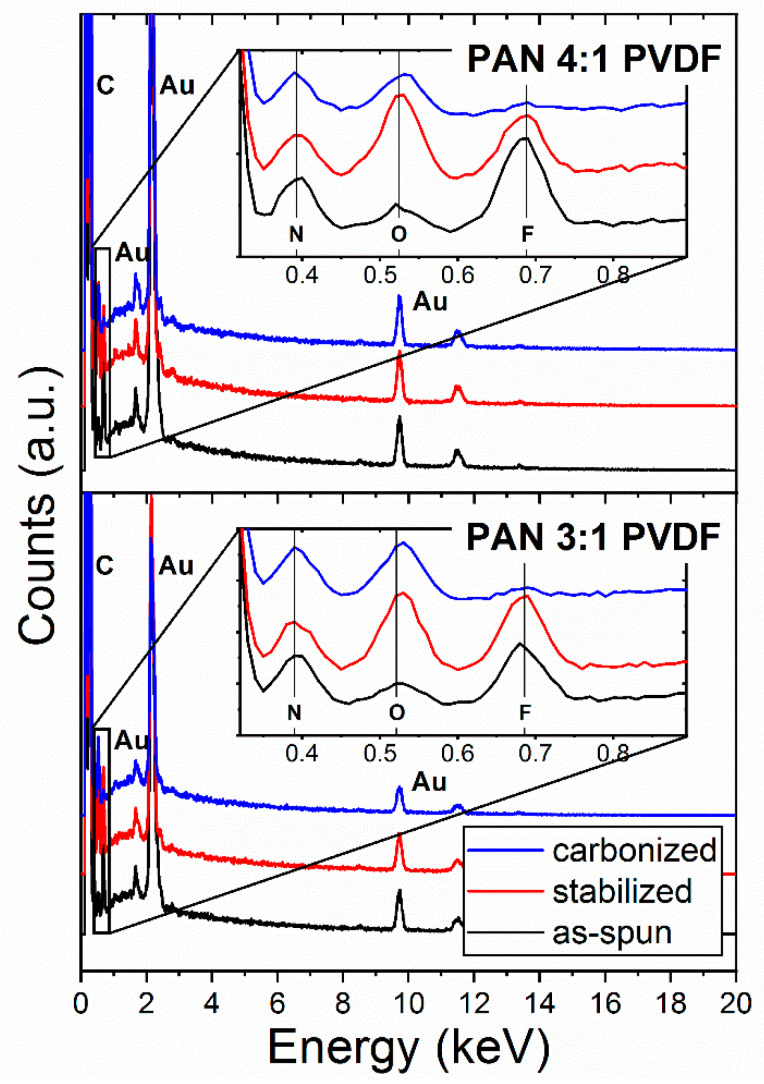
Normalized EDX spectra of as-spun, stabilized and carbonized blend nanofibers.

After stabilization, it can be seen that the surface of the PVDF-modified fibers has a significantly lower proportion of nitrogen than the pure PAN, which is due to the reduced relative PAN concentration. The fluorine and oxygen concentration in the surface of those fibers, however, increase significantly (see Figure 7b: stabilized 3:1 and 4:1). One explanation for this could be that the yet mostly unaffected PVDF polymers are displaced from the core of the fiber during the formation of the PAN ladder-structure and diffuse to the fiber surface. In the subsequent carbonization, these would mostly decompose, as seen in the TGA measurements. Reactive fluorine compounds of lower molecular weight such as HF, C_2_H_2_F_2_ and C_4_H_3_F_3_ as decomposition products [76] either are desorbed or react with the ladder-polymer at the fiber surface. This assumption would also be consistent with the observation that, although the XPS measurements show a substantial fluorine concentration in the fiber surface (see Figure 7b: carbonized 3:1 and 4:1, F 1s), the EDX measurements shown in Figure 8 for the bulk material contain a barely noticeable fluorine peak after carbonization.

Surprisingly, the pure PAN fibers, which showed a significantly higher proportion of oxygen before stabilization, show a strong decrease after stabilization, which may be explained by the significant increase in the fiber diameter. The oxygen content in the surface of blend fibers, however, increased as expected. Of course, it should be noted here that the presence of hydrogen is not taken into account. From other studies, it is known that at 500 °C more than 3% hydrogen can still be expected, which only escapes at significantly higher temperatures [92].

Fluorine is detectable in all stages of treatment. However, the relative ratio between the 3:1 and 4:1 samples does not correspond to what one would expect from the concentration ratios in the polymer solution. These inconsistencies on the one hand reflect the already mentioned challenges of XPS analysis in general and on the other hand the non-negligible influence of the nonwoven morphology. While the fiber diameter and pore size influence the specific surface area and thus adsorption and desorption processes, the nonwoven thickness determines the proportion of fibers that are in direct contact with the atmosphere during treatment. In view of the standard deviation in the thickness measurement and possibly inhomogeneous distribution of gas flow and temperature in the treatment furnace, the results are expected to be associated with a certain variance.

Since the sensitivity of EDX spectroscopy is not well suited for the analysis of light elements [93], a quantitative evaluation of the spectra shown in Figure 8 is probably not reliable. Nevertheless, EDX spectra show similar qualitative elemental compositions as XPS spectra. As expected, the EDX spectra show an increase in oxygen after oxidative stabilization. As stated above, in contrast to the XPS spectra, the N:F ratio does not appear to have changed significantly after stabilization, indicating that the PVDF polymers diffused to the fiber surface during stabilization. During carbonization, however, the fluorine content decreases significantly. While the XPS data still show a considerable amount of fluorine, the fluorine peaks in the EDX spectra are hardly noticeable. It can therefore be assumed that remaining fluorine compounds are mainly located on the fiber surface.As the carbonization temperature increases, a steady loss of fluorine content would be expected. Shi et al. confirmed this at higher carbonization temperatures for pure PVDF between 600 and 1000 °C [62].

In the as-spun state, the two polymers are not covalently bonded together. Whether or how the two polymers built a mutual covalently bonded molecular structure during carbonization and what form the remaining fluorine compounds ultimately adopted remains largely open.

### 3.4. Electrical Properties

**Figure 9:**
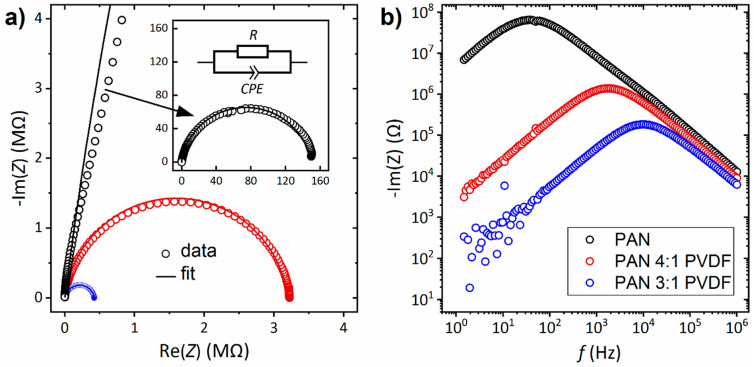
Impedance spectra of all carbonized nanofibers at 25 °C: (**a**) imaginary part vs. real part (Nyquist plot); and (**b**) imaginary plot vs. frequency (Bode plot).

Lastly, impedance measurements were carried out to investigate the electrical conductivity of the nanofibers. Figure 9 shows spectra of three samples, prepared from pure PAN and from PAN/PVDF blends. The Nyquist plots (imaginary part vs. real part of the complex impedance) exhibit depressed arcs that can be modeled by a circuit equivalent of a resistor and a parallel constant phase element (CPE), as indicated in the figure. From the resistance R, the conductivity is calculated by

(2) $$\sigma =\frac{d}{R\;A}$$

where d is the average nonwoven thickness and A is the cross-sectional area, corresponding to the contact area between the electrodes and the sample [94]. All three samples exhibit only negligible conductivity (Table 1), which indicates that the low carbonization temperature of 500 °C does not lead to sufficient formation of conducting percolation paths along the CNFs. It has been shown, however, that the conductivity is increased by up to two orders of magnitude by blending PAN with PVDF. This can be explained on the one hand by the increased fiber diameter, which leads to increased electrically conducting percolation paths. On the other hand, it is known that conductivity increases with a higher fluorine content [95,96]. In the literature, carbon nanofibers prepared from electrospun PAN without further components showed conductivities in the range 10–20,000 S/m, yet these materials were carbonized at high temperatures of 800 °C to 1000 °C [26,37,97,98,99]. The wide range of reported conductivities can be attributed to different stabilization and carbonization methods. Decisive factors are heating rate, final temperature and holding time, as well as spatial arrangement, atmosphere and fiber alignment. As a result, the values shown in Table 1 are difficult to compare with the literature.

## 4. Conclusions

PAN/PVDF blend nanofibers were produced from a binary DMSO polymer solution using a wire-based electrospinning process. Subsequent oxidative stabilization up to 280 °C and incipient carbonization up to 500 °C were performed on the as-spun blend nanofibers. The chemical and morphological changes in the blended nanofibers after the thermal treatment steps were investigated with the aid of various characterization methods. It was shown that the blend nanofibers are thicker and more inhomogeneous in the as-spun state and show better dimensional stability during thermal treatment compared to pure PAN. With the help of TGA, the contribution of the respective polymers to the total nonwoven mass during each stage of treatment could be calculated. DSC measurements show a reduced degree of crystallinity of the electrospun PVDF compared to the raw material. With the help of FTIR, XPS, EDX and NMR, it could be shown that PAN undergoes the well-established transition towards a ladder-polymer structure during stabilization. A comparative analysis of all measurements suggests that, during stabilization, the PVDF polymer, which has not been affected until that point (as shown by TGA), diffuses to the fiber surface (as shown by an increased XPS fluorine signal in contrast to a constant EDX signal). It then decomposes there during the incipient carbonization (as shown by TGA and NMR) and then partially remains in the fiber surface (as shown by the XPS and EDX). The remaining fluorine content of the resulting CNF is also reflected in a significant increase in conductivity with increasing PVDF concentration in the initial spinning-solution, an effect, which is stronger for the 3:1 blend than for the 4:1 blend, making the former more suitable for applications, in which conductivity is relevant. Further investigations will show at which carbonization temperatures the fluorine content will be fully decomposed. In addition, investigations applying previous chemical stabilization of the PVDF component can be used to investigate whether the overall carbon yield is increased in this case, taking into account that, while studies of other groups show that chemical stabilization is not necessary, it may still be useful to optimize the carbonization result.

The investigations contribute to a better understanding of the molecular processes during the thermal treatment of blend nanofibers and exemplify possibilities and challenges in material analysis with regard to these processes. The enrichment of CNF with desired constituents by spinning binary polymer solutions could represent an efficient alternative to the subsequent doping of nanofibers by post-treatment, thus enhancing their functionality in electronic devices and other applications.

## Figures and Tables

**Table 1:** Exact thickness values as shown in Figure 3 for all nonwovens as well as resistances and conductivities of all carbonized nonwovens at 25 °C.

Sample	Thickness d (µm)			Resistance R (MΩ)	Conductivity σ (S/m)
	As-Spun	Stabilized	Carbonized		
PAN	15.4 ± 4.4	21.7 ± 5.2	22.3 ± 4.4	148	5.9 × 10^−10^
PAN 4:1 PVDF	3.8 ± 1.0	5.3 ± 1.5	5.4 ± 1.1	3.20	5.4 × 10^−9^
PAN 3:1 PVDF	11.5 ± 2.0	13.0 ± 3.0	13.1 ± 2.5	0.42	9.9 × 10^−8^
